# Supplementary material for: Allosteric regulation and crystallographic fragment screening of SARS-CoV-2 NSP15 endoribonuclease
Source: Nucleic Acids Res. 2023 Apr 28;51(10):5255–70. doi: 10.1093/nar/gkad314 (PMC10250223; doi:10.1093/nar/gkad314)
Supplement: gkad314_Supplemental_Files [file gkad314_supplemental_files.zip › Supplementary datasets.zip!/159/Refine_4/01-BUSTER/Cycle-2/shell.01/R_Rfree.html]

# Rxpct factors in resolution bins at cycle 0


|  |  |  |  |  |  |  |  |  |
| --- | --- | --- | --- | --- | --- | --- | --- | --- |
|  | **<d> (Å)**  Rxpct\_Work | Rxpct\_Free | Rxpct\_All | |  |  |  |  |
|  | 12.8357 | 0.3437 | 0.4282 | 0.3472 |  |
|  | 7.4107 | 0.2982 | 0.3186 | 0.2994 |  |
|  | 5.7403 | 0.2736 | 0.2889 | 0.2742 |  |
|  | 4.8514 | 0.1853 | 0.1943 | 0.1858 |  |
|  | 4.2786 | 0.1693 | 0.1659 | 0.1691 |  |
|  | 3.8701 | 0.1885 | 0.2007 | 0.1891 |  |
|  | 3.5600 | 0.2034 | 0.2179 | 0.2041 |  |
|  | 3.3142 | 0.2087 | 0.2307 | 0.2100 |  |
|  | 3.1131 | 0.1980 | 0.2098 | 0.1985 |  |
|  | 2.9447 | 0.1955 | 0.2077 | 0.1960 |  |
|  | 2.8010 | 0.1896 | 0.1835 | 0.1893 |  |
|  | 2.6764 | 0.1854 | 0.1969 | 0.1860 |  |
|  | 2.5671 | 0.1940 | 0.1962 | 0.1941 |  |
|  | 2.4702 | 0.1978 | 0.1980 | 0.1978 |  |
|  | 2.3835 | 0.1958 | 0.2020 | 0.1961 |  |
|  | 2.3054 | 0.1896 | 0.2122 | 0.1906 |  |
|  | 2.2344 | 0.1973 | 0.2130 | 0.1981 |  |
|  | 2.1696 | 0.2004 | 0.2061 | 0.2007 |  |
|  | 2.1102 | 0.2155 | 0.2085 | 0.2152 |  |
|  | 2.0554 | 0.2389 | 0.2254 | 0.2382 |  |
|  | **Overall** | 0.2104 | 0.2216 | 0.2110 |  |

# Rxpct factors in resolution bins at cycle 1


|  |  |  |  |  |  |  |  |  |
| --- | --- | --- | --- | --- | --- | --- | --- | --- |
|  | **<d> (Å)**  Rxpct\_Work | Rxpct\_Free | Rxpct\_All | |  |  |  |  |
|  | 12.8357 | 0.3439 | 0.4283 | 0.3475 |  |
|  | 7.4107 | 0.2985 | 0.3188 | 0.2997 |  |
|  | 5.7403 | 0.2739 | 0.2879 | 0.2745 |  |
|  | 4.8514 | 0.1864 | 0.1954 | 0.1869 |  |
|  | 4.2786 | 0.1707 | 0.1667 | 0.1704 |  |
|  | 3.8701 | 0.1895 | 0.2024 | 0.1901 |  |
|  | 3.5600 | 0.2046 | 0.2208 | 0.2054 |  |
|  | 3.3142 | 0.2105 | 0.2339 | 0.2119 |  |
|  | 3.1131 | 0.2015 | 0.2120 | 0.2019 |  |
|  | 2.9447 | 0.1988 | 0.2130 | 0.1994 |  |
|  | 2.8010 | 0.1941 | 0.1887 | 0.1938 |  |
|  | 2.6764 | 0.1887 | 0.1999 | 0.1893 |  |
|  | 2.5671 | 0.1940 | 0.1978 | 0.1943 |  |
|  | 2.4702 | 0.1971 | 0.1942 | 0.1969 |  |
|  | 2.3835 | 0.1944 | 0.2014 | 0.1947 |  |
|  | 2.3054 | 0.1879 | 0.2127 | 0.1891 |  |
|  | 2.2344 | 0.1968 | 0.2113 | 0.1974 |  |
|  | 2.1696 | 0.2004 | 0.2068 | 0.2007 |  |
|  | 2.1102 | 0.2160 | 0.2097 | 0.2157 |  |
|  | 2.0554 | 0.2404 | 0.2271 | 0.2397 |  |
|  | **Overall** | 0.2116 | 0.2231 | 0.2122 |  |

# Rxpct factors in resolution bins at cycle 2


|  |  |  |  |  |  |  |  |  |
| --- | --- | --- | --- | --- | --- | --- | --- | --- |
|  | **<d> (Å)**  Rxpct\_Work | Rxpct\_Free | Rxpct\_All | |  |  |  |  |
|  | 12.8357 | 0.3443 | 0.4291 | 0.3479 |  |
|  | 7.4107 | 0.2987 | 0.3188 | 0.2999 |  |
|  | 5.7403 | 0.2743 | 0.2882 | 0.2749 |  |
|  | 4.8514 | 0.1872 | 0.1963 | 0.1877 |  |
|  | 4.2786 | 0.1717 | 0.1671 | 0.1714 |  |
|  | 3.8701 | 0.1901 | 0.2035 | 0.1908 |  |
|  | 3.5600 | 0.2052 | 0.2228 | 0.2060 |  |
|  | 3.3142 | 0.2113 | 0.2353 | 0.2127 |  |
|  | 3.1131 | 0.2030 | 0.2128 | 0.2034 |  |
|  | 2.9447 | 0.2000 | 0.2152 | 0.2007 |  |
|  | 2.8010 | 0.1953 | 0.1914 | 0.1951 |  |
|  | 2.6764 | 0.1893 | 0.2003 | 0.1899 |  |
|  | 2.5671 | 0.1938 | 0.1978 | 0.1940 |  |
|  | 2.4702 | 0.1962 | 0.1928 | 0.1960 |  |
|  | 2.3835 | 0.1935 | 0.2008 | 0.1938 |  |
|  | 2.3054 | 0.1873 | 0.2126 | 0.1885 |  |
|  | 2.2344 | 0.1964 | 0.2105 | 0.1970 |  |
|  | 2.1696 | 0.2003 | 0.2079 | 0.2006 |  |
|  | 2.1102 | 0.2156 | 0.2105 | 0.2154 |  |
|  | 2.0554 | 0.2398 | 0.2269 | 0.2391 |  |
|  | **Overall** | 0.2122 | 0.2239 | 0.2128 |  |

# Rxpct factors in resolution bins at cycle 3


|  |  |  |  |  |  |  |  |  |
| --- | --- | --- | --- | --- | --- | --- | --- | --- |
|  | **<d> (Å)**  Rxpct\_Work | Rxpct\_Free | Rxpct\_All | |  |  |  |  |
|  | 12.8357 | 0.3227 | 0.4054 | 0.3261 |  |
|  | 7.4107 | 0.2750 | 0.3123 | 0.2773 |  |
|  | 5.7403 | 0.2801 | 0.3238 | 0.2818 |  |
|  | 4.8514 | 0.2171 | 0.2434 | 0.2185 |  |
|  | 4.2786 | 0.2097 | 0.2024 | 0.2093 |  |
|  | 3.8701 | 0.2174 | 0.2396 | 0.2185 |  |
|  | 3.5600 | 0.2298 | 0.2652 | 0.2315 |  |
|  | 3.3142 | 0.2354 | 0.2732 | 0.2377 |  |
|  | 3.1131 | 0.2311 | 0.2396 | 0.2315 |  |
|  | 2.9447 | 0.2174 | 0.2229 | 0.2176 |  |
|  | 2.8010 | 0.2103 | 0.2157 | 0.2106 |  |
|  | 2.6764 | 0.2003 | 0.2155 | 0.2011 |  |
|  | 2.5671 | 0.1926 | 0.1968 | 0.1929 |  |
|  | 2.4702 | 0.1860 | 0.1905 | 0.1862 |  |
|  | 2.3835 | 0.1828 | 0.1978 | 0.1834 |  |
|  | 2.3054 | 0.1737 | 0.1924 | 0.1746 |  |
|  | 2.2344 | 0.1848 | 0.2020 | 0.1856 |  |
|  | 2.1696 | 0.1887 | 0.1936 | 0.1890 |  |
|  | 2.1102 | 0.1990 | 0.2028 | 0.1992 |  |
|  | 2.0554 | 0.2235 | 0.2130 | 0.2229 |  |
|  | **Overall** | 0.2246 | 0.2444 | 0.2256 |  |
